# Effectiveness of balance training on pain and functional outcomes in knee osteoarthritis: A systematic review and meta-analysis

**DOI:** 10.12688/f1000research.111998.1

**Published:** 2022-06-01

**Authors:** Ashish John Prabhakar, Shruthi R, Dias Tina Thomas, Pradeepa Nayak, Abraham M. Joshua, Srikanth Prabhu, Yogeesh Dattakumar Kamat

**Affiliations:** 1 Department of Physiotherapy, Kasturba Medical College, Mangalore, Manipal Academy of Higher Education, Manipal, India; 2 Department of Computer Science and Engineering, Manipal Institute of Technology, Manipal Academy of Higher Education, Manipal, India; 3 Consultant Knee & Hip Surgeon, Department of Orthopedics, Kasturba Medical College, Magalore, Manipal Academy of Higher Education, Manipal, India

**Keywords:** knee osteoarthritis, balance assessment, proprioception, exercise therapy, visual reality

## Abstract

**Background:** Knee osteoarthritis (OA) is a musculoskeletal disorder that causes pain and increasing loss of function, resulting in reduced proprioceptive accuracy and balance. Therefore, the goal of this systematic review and meta-analysis is to evaluate the effectiveness of balance training on pain and functional outcomes in knee OA.

**Methods:** “PubMed”, “Scopus”, “Web of Science”, “Cochrane”, and “Physiotherapy Evidence Database” were searched for studies conducted between January 2000 and December 2021. Randomized controlled trials (RCTs) that investigated the effectiveness of balance training in knee OA, as well as its effects on pain and functional outcome measures, were included. Conference abstracts, case reports, observational studies, and clinical commentaries were not included. Meta-analysis was conducted for the common outcomes, i.e., Visual Analog Scale (VAS), The Timed Up and Go (TUG), Western Ontario and McMaster Universities Arthritis Index (WOMAC). The PEDro scale was used to determine the quality of the included studies.

**Results:** This review includes 22 RCTs of which 17 articles were included for meta-analysis. The included articles had 1456 participants. The meta-analysis showed improvement in the VAS scores in the experimental group compared to the control group [ *I* ^2^= 92%; mean difference= -0.79; 95% CI= -1.59 to 0.01; p<0.05] and for the WOMAC scores the heterogeneity ( *I* ^2^) was 81% with a mean difference of -0.02 [95% CI= -0.44 to 0.40; p<0.0001]. The TUG score was analyzed, the *I* ^2^ was 95% with a mean difference of -1.71 [95% CI= -3.09 to -0.33; p<0.0001] for the intervention against the control group.

**Conclusions:** Balance training significantly reduced knee pain and improved functional outcomes measured with TUG. However, there was no difference observed in WOMAC. Although due to the heterogeneity of the included articles the treatment impact may be overestimated.

**Registration:** The current systematic review was registered in PROSPERO on 7th October 2021 (registration number CRD42021276674).

## Introduction

Knee osteoarthritis (OA) is a common disease in older adults that causes chronic disability. ^1^ With age the occurrence of knee OA increases, with rates of approximately 13% among women and 10% among men aged 60 years and above. Pathology is associated with changes in cartilage, bones, surrounding soft tissues, and muscles. Patients with knee OA gradually lose function, indicating an increased reliance while climbing stairs and walking, and demonstrate an increased dependency in functional tasks. ^2^ Balance forms a fundamental component of many of these activities. ^3^ Balance is a complex function of multiple neuromuscular systems, which includes sensory, motor and integrative components. In older adults, impaired balance is linked to the risks of falls and diminished mobility.

Falls have been shown to be more frequent during activities that require relocation of the body’s center of mass (COM), such as ascending and descending stairs and walking. ^4^^,^^5^ According to existing data, older adults with bilateral or unilateral knee OA exhibit lower postural stability. Understanding the influence of knee OA on balance may aid in the identification of potential impairment pathways in these individuals, permitting for a more comprehensive disease management. ^3^

The functional knee joint is subjected to continual strain. Though the active muscle contraction and bone geometry offer stability to the knee joint during normal daily activities and the mechanoreceptors help to maintain stability by providing sensory feedback that facilitates antagonist-agonist muscle activity, ^6^ alterations in joint kinematics as a result of disease processes can have a direct impact on balance control and gait parameters. Proprioceptive insufficiency can be caused by pain, inflammation or mechanical stress. This impairment has been linked to aberrant pressure buildup in the surrounding tissues, which prevents the influx of sensorimotor information regarding joint position sense (JPS) and movements. ^7^ Such inadequacies affect the dynamic stability provided by the muscles surrounding the joint, likely to result in functional instability. ^8^

Conservative treatment is recommended to relieve symptoms and enhance functional activity performance, and may prevent muscle weakness and thereby halt disease progression. ^8^ As per the existing literature, many types of exercises have been undertaken to improve proprioception and balance in knee osteoarthritis. Walking, retro walking, kinesthetic balancing, closed kinetic chain and aerobic dance, have all been shown to improve proprioception and balance function. The majority of past research involved individuals with mild to intermediate stages of knee OA, while some patients with advanced stages were also involved. Despite the presence of literature on the effectiveness of the various forms of exercises in individuals with knee OA, no systematic review, to the best of our knowledge, describes the effectiveness of various balance exercise strategies. Therefore, the purpose of this systematic review is to examine and identify the available information on the impact of various balance training strategies on pain and functional outcomes in people with knee OA.

## Methods

The current systematic review was registered in PROSPERO with the registration number CRD42021276674. The Preferred Reporting Items for Systematic Reviews and Meta-Analyses (PRISMA) criteria were followed throughout the review procedure. The key search words and completed PRISMA checklist can be found as *Extended data.* ^43^

### Eligibility criteria

*Inclusion criteria*

1) Randomized controlled trials (RCTs) comparing balance exercises to conventional exercises or no therapy and examining functional results in participants with knee OA were examined.
2) Participants in the chosen studies could be either male or female, of any age or severity level.
3) Articles with a PEDro score ^9^ of five or above were considered.

*Exclusion criteria*

1) Conference abstracts, case reports, observational studies, and clinical commentaries were not included.
2) Systemic arthritic illnesses, tibial osteotomy, hip or knee joint replacement, and any other muscle or neurological ailment that may affect the lower extremity and impair balance were excluded.

### Search strategy

“ PubMed” (PubMed, RRID:SCR_004846), “ Scopus”, “ Web of Science”, “ Cochrane” (Cochrane Library, RRID:SCR_013000) and “ PEDro” were searched in January 2022 for relevant articles that established the efficacy of various methods of balance training for participants with knee OA. The studies featured were written in English. Two independent investigators conducted the search, which included a combination of two primary keywords: “Knee OA” (population) AND “Balance.” The Boolean operators “AND” and “OR” were used to combine the two terms. The search techniques were adjusted based on the databases. The publication dates were not confined, and the review included works published between January 2000 and December 2021.

### Study selection criteria

Conference abstracts, case reports, observational studies, and clinical commentaries were not included. Systemic arthritic illnesses, tibial osteotomy, hip or knee joint replacement, and any other muscle or neurological ailment that may affect the lower extremity and impair balance were excluded. RCTs comparing balance exercises to conventional exercises or no therapy and examining functional results in participants with knee OA were examined. Participants in the chosen studies could be either male or female, of any age or severity level. Articles with a PEDro score ^9^ of five or above were considered. Two independent reviewers screened the articles to check if they met the inclusion criteria, conflicts between the reviewers was resolved by an intervening third reviewer.

### Study risk of bias assessment

The included articles were RCTs, therefore the risk of bias was done using the PEDro score. The PEDro scoring system consists of a checklist of 10 scored yes-or-no questions pertaining to the internal validity and the statistical information provided. PEDro scores of 0-3 were considered ‘poor’, 4-5 ‘fair’, 6-8 ‘good’, and 9-10 ‘excellent’.

### Effect measures

Standard mean difference was used as an effect measure for comparison between balance treatment and routine rehabilitation.

### Synthesis methods

The search was conducted by two independent reviewers (DT, SR) on various databases, following which all the identified studies were imported into online software Rayyan QCRI (Rayyan QCRI, RRID:SCR_017584). The titles and abstracts were also screened by two reviewers. For any ambiguities in the studies, consensus was obtained by discussing with the third reviewer (AP). The eligibility assessment under the inclusion-exclusion criteria was carried out by reviewing full-text articles.

The first reviewer obtained data from the included articles, which was then substantiated by a second reviewer and were entered into a standard form developed for the review. Information about the authors, journal, year of publication, characteristics of the subjects (age, inclusion criteria, gender, sample size), method (i.e., design, subjects, intervention, measures), outcome assessed, details of the interventions (parameters, frequency, intensity, type, time) and comparison groups were extracted from the included articles. Quantitative analysis, for the homogenous outcomes, i.e., VAS, TUG and WOMAC, was done. The pooled estimates of effect size were calculated using the effects model. These pooled estimations will subsequently be depicted in forest plots.

### Quality assessment

The first two reviewers independently completed a procedural quality assessment of the studies based on the PEDro scale, and papers with a PEDro score of less than five were eliminated. The fourth reviewer resolved any doubts about the study’s quality (SP). Each question on the PEDro scale evaluates the statistical significance and internal validity of the trials. Studies having a score of greater than five out of 10 ( Table 1) were determined to have high procedural quality, and the study included 22 articles.

**Table 1.** PEDro scoring.

Trial	1	2	3	4	5	6	7	8	9	10	11	Score
1. Chen *et al*. ^19^	✓	✓	✓	✓	✗	✗	✓	✓	✓	✓	✓	8/10
2. Jahanjoo *et al*. ^17^	✓	✓	✗	✓	✗	✗	✓	✓	✓	✓	✓	7/10
3. Ojoawo *et al*. ^27^	✓	✓	✓	✓	✗	✗	✗	✓	✗	✓	✓	6/10
4. Gomiero *et al*. ^11^	✓	✓	✓	✓	✗	✗	✗	✓	✓	✓	✓	7/10
5. Fitzgerald *et al*. ^12^	✓	✓	✓	✓	✗	✗	✓	✓	✓	✓	✓	8/10
6. Xiao *et al*. ^13^	✓	✓	✗	✓	✗	✗	✗	✓	✗	✓	✓	5/10
7. Xiao *et al*. ^14^	✓	✓	✓	✓	✗	✗	✓	✓	✗	✓	✓	7/10
8. Cho *et al*. ^18^	✓	✓	✓	✓	✗	✗	✗	✗	✗	✓	✓	5/10
9. Jan *et al*. ^41^	✓	✓	✗	✗	✗	✗	✓	✓	✗	✓	✓	5/10
10. Hiyama *et al*. ^32^	✓	✓	✓	✓	✗	✗	✗	✓	✗	✓	✓	6/10
11. Hussein *et al*. ^24^	✓	✓	✓	✓	✗	✗	✗	✗	✗	✓	✓	5/10
12. Kuru Çolak *et al*. ^23^	✓	✓	✓	✓	✗	✗	✗	✗	✓	✓	✓	6/10
13. UzunkulaoĞlu *et al*. ^31^	✓	✓		✓	✗	✗	✗	✓	✗	✓	✓	5/10
14. Oh *et al*. ^15^	✓	✓	✓	✓	✗	✗	✓	✓	✓	✓	✓	8/10
15. Tunay *et al*. ^20^	✓	✓	✗	✓	✗	✗	✗	✓	✗	✓	✓	5/10
16. Reza *et al*. ^21^	✓	✓	✗	✓	✗	✓	✗	✓	✗	✓	✓	6`/10
17. Rogers *et al*. ^29^	✓	✓	✗	✓	✓	✗	✗	✗	✗	✓	✓	5/10
18. Lin *et al*. ^16^	✓	✓	✓	✓	✗	✗	✗	✓	✗	✓	✓	5/10
19. Rahlf *et al*. ^30^	✓	✓	✓	✓	✗	✗	✗	✓	✓	✓	✓	7/10
20. Trans *et al*. ^26^	✓	✓	✓	✓	✗	✗	✗	✓	✓	✓	✓	7/10
21. Braghin *et al*. ^25^	✓	✓	✓	✓	✗	✗	✗	✓	✓	✓	✓	7/10
22. Jan *et al*. ^33^	✓	✓	✗	✓	✗	✗	✓	✓	✓	✓	✓	7/10

## Results

### Description of studies

**Figure 1.**
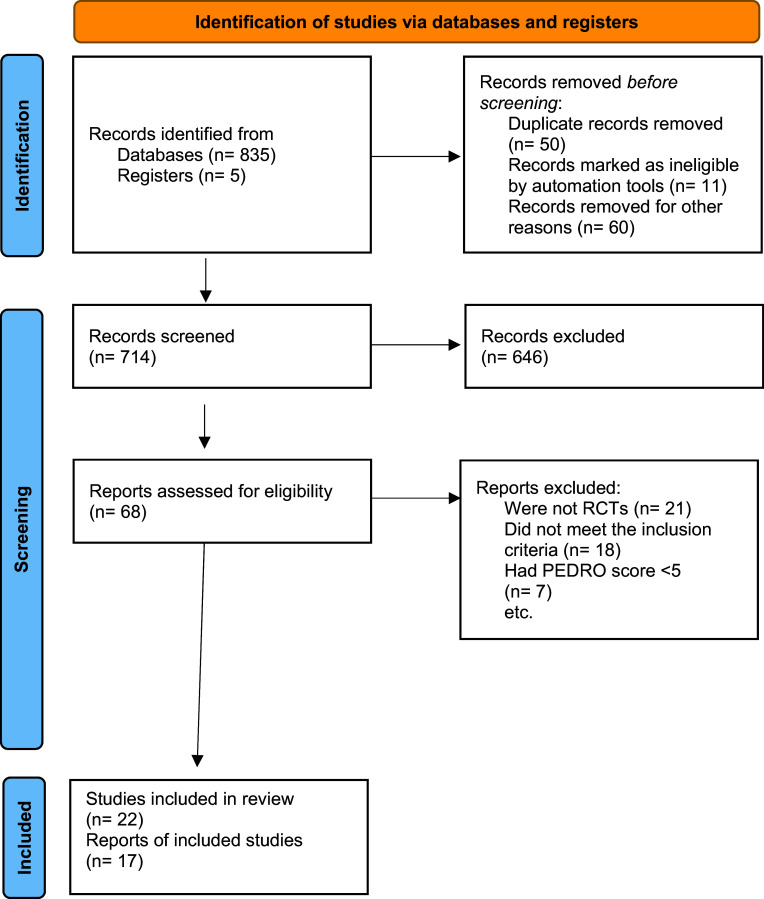
PRISMA flowchart. PRISMA, Preferred Reporting Items for Systematic Reviews and Meta-Analyses; RCT, Randomized controlled trial.

*Search results:* We identified 22 studies for the systematic review and 17 studies for the meta-analysis out of 835 identified through the database screening. The results of the search and selection process are presented in the PRISMA flow diagram ( Figure 1). The included articles were published between January 2000 and December 2021.

*Study designs:* All the included articles were RCTs.

*Participants:* A total of 1456 people were involved in the included studies. The participants ranged in age from 30 to 65 years. The study encompassed all Kellgren Lawrence severity levels of knee OA. ^10^

*Intervention:* The intervention group in eight of the 22 trials received isolated balance training programs with no other form of exercise. Whereas 14 out of the 22 studies provided balance training along with some other form of exercises. Two trials provided agility training to the experimental group, ^11^^,^^12^ whereas the experimental group in the other two studies received Wu Qin xi Qigong and Wuqinxi exercise programs. ^13^^,^^14^ Two trials used virtual reality and virtual feedback to give balance training, ^15^^–^^17^ while the third employed Kinesio tapping to improve stability in subjects with knee OA. ^18^ In one study, body weight (BW) training was paired with needle knife therapy as a treatment modality. ^19^

*Control intervention:* The participants in the control groups received routine rehabilitation and strengthening exercises.

*Outcomes:* The included studies analyzed the following outcomes: VAS, ^11^^,^^15^^,^^17^^,^^20^^–^^24^ WOMAC, TUG,  self-reported knee joint instability, Berg Balance Scale (BBS), Lequesne index, knee joint proprioception, Tinetti’s Performance Oriented Mobility Assessment (POMA) scale and static postural stability. Eight of the included studies analyzed pain using the VAS, ^11^^,^^15^^,^^17^^,^^20^^–^^24^ and 12 studies analyzed the function using WOMAC score. ^11^^–^^13^^,^^15^^,^^17^^,^^19^^,^^20^^,^^25^^–^^30^ TUG score was analyzed by six studies. ^11^^,^^14^^,^^17^^,^^20^^,^^31^^–^^33^

*Study characteristics:* The size of the samples ranged from 30 to 190 subjects. All the subjects included in the trial were analyzed based on the American College of Rheumatology criteria. ^34^ All the studies used the Kellgren Lawrence score for radiological grading.

### Effect of intervention

The effects of interventions are explained in Table 2.

**Table 2.** Effects of intervention of the included studies.

Author	Samples	Results	Outcomes	Experimental group		Control group	
				Pre-intervention	Post-intervention	Pre-intervention	Post-intervention
1. Reza *et al*., 2018 ^21^	30 subjects Intervention Group (n = 15) Control Group (n = 15)	There was no significant difference in mean pain score between the balancing exercises and the control group, however balance exercises enhanced self-reported knee joint instability score when compared to a control group.	VAS	6.53±2.32	4.60±1.91	7.46±1.92	5.40±2.09
			Self-reported knee joint instability	1.80±0.94	2.93±0.79	1.40±0.73	2.13±1.06
2. Tunay *et al*., 2010 ^20^	60 subjects Intervention Group (n = 30) Control Group (n = 30)	Both hospital-based and home-based exercises decreased joint pain and functional status in people with knee OA.	VAS	5.60±2.31	1.46±2.04	5.96±2.14	2.80±2.02
			WOMAC	10.22±4.51	5.45±3.76	9.48±3.61	5.69±2.84
			System-proprioceptive test	11.96±2.10	14.26±2.88	12.33±2.59	13.03±2.97
			TUG	6.25±1.33	5.19±1.05	6.85±1.84	5.39±1.46
3. Oh *et al*., 2020 ^15^	26 subjects Intervention Group (n = 13) Control Group (n = 13)	Knee joint muscle-strengthening exercise combined with visual information feedback training benefited patients with degenerative knee arthritis and improve their balance function and pain. This training technique is thought to help people with progressive knee arthritis improve their upright balance and reduce pain.	VAS	6.9±1.8	4.2±1.7	7.0±2.0	4.9±1.4
			WOMAC	33.3±13.7	15.7±8.7	28.5±10.6	22.9±8.3
4. UzunkulaoĞlu *et al*., 2020 ^31^	50 subjects Intervention Group (n = 25) Control Group (n = 25)	Both single-task and dual-task training improve balance function in older patients with knee OA. Dual-task training does not outperform single-task training when it comes to improving balance in older people with OA.	BBS	29.4±5.4	34.8±5.7	30.5±6.1	34.5±6.3
			KAT 2000 static score	1528.4±474.1	1232.9±487.5	1468.1±466.6	1167.3±468.0
			KAT 2000 dynamic score	2020.6±441.7	1567.8±455.0	2006.6±447.4	1683.8±450.0
			TUG single task	13.7±4.4	11.6±4.3	13.2±4.3	11.2±4.3
			TUG dual task	15.8±5.1	12.6±5.0	15.3±5.1	14.3±5.1
5. Kuru Çolak *et al*., 2017 ^23^	78 Subjects Intervention Group (n = 33) Control Group (n = 23)	Low-intensity lower extremity exercises performed in a clinic under the supervision of a physiotherapist were found to be more effective than home-based exercises in reducing post-activity discomfort and increasing quadriceps hamstring strength.	VAS	67.61±4.655	39.58±4.39	62.61±5.90	50.09±6.99
			6MWT	352±19.65	382±15.7	381±24.2	411±24.7
			Balance score	80.7±1.85	83.3±2.6	82.7±4.7	81.6±4.2
6. Hussein *et al*., 2015 ^24^	59 subjects Intervention Group (n = 38) Control Group (n = 21)	Adding balance training to resistive exercise improves muscular strength, functional status and knee postural control accuracy in people with knee OA.	VAS	81.47±10.98	40±14.23	72.8±17.07	42.8±15.21
			Lequesne index	8.79±0.41	6.42±0.068	8.71±0.46	6.43±0.507
			Knee proprioception				
			10	24.631±45.06	4.578±8.416	35.714±30.71	17.14±15.21
			30	14.379±14.541	4.38±5.476	25.71±12.345	8.57±12.089
			60	11.896±8.738	4.729±4.811	7.85±11.056	1.237±2.376
7. Hiyama *et al*., 2012 ^32^	40 subjects Intervention Group (n = 20) Control Group (n = 20)	According to the findings of this study, walking exercise improves dual task performance and executive function in patients with knee OA.	TUG	12.9±2.0	12.0±1.5	13.0±2.1	13.0±2.2
			Tandem gait	12.9±1.8	11.8±1.4	13.4±1.7	13.3±12
			TMT	63.4±43.1	48.3±29.6	58.1±37.6	60.3±30.7
8. Jan *et al*., 2008 ^41^	49 subjects Intervention Group (n = 24) Control Group (n = 25)	TMFSE in sitting appears to be an alternative for exercise in people with mild to moderate knee OA. This may be a particularly enticing option for folks who feel pain when conducting weight-bearing exercises.	Ground level walking	44.1±2.9	38.6±2.5	41.7±3.1	42.3±2.6
			Stairs	34.2±2.1	26.5±2.3	32.2±2.3	33.1±3.4
			Figure-of-eight	51.3± 6.7	29.1±3.6	38.4±3.8	39.8±4.8
9. Cho *et al*., 2015 ^18^	46 subjects Intervention Group (n = 23) Control Group (n = 23)	These findings imply that applying sufficient stress to the quadriceps successfully reduces various forms of pain while improving AROM and proprioception in OA patients. As a result, KT may be a useful intervention in clinics for relieving pain, improving AROM, and improving proprioception.	VAS	67.2±9.1	50.0±7.7	68.4±7.1	67.2±7.2
			Proprioception				
			15	10.3±3.72	3.3±1.06	10.2±3.42	9.4±3.29
			30	11.9±4.62	3.2±1.97	11.9±4.73	11.3±3.98
			45	14.5±3.50	3.2±1.37	14.4±7.24	13.9±5.50
10. Xiao *et al*., 2020 ^14^	98 subjects Intervention Group (n = 40) Control Group (n = 45)	WQXQ and regular physical therapy exercise routines were both significantly beneficial in reducing activity limits and pain while also enhancing balance and muscle power. WQXQ was proven to be more effective than typical physical therapy exercise in improving balance and lowering pain in those with knee OA.	WOMAC	28.9±11.7	20.7±8.7	27.4±10.9	18.8±7.4
			TUG	9.2±2.2	7.7±2.7	9.9±2.9	7.9±2.2
			6MWT	359.3±62.7	405.2±68.9	361.8±66.9	411.2±67.6
			BBS	39.9±6.4	45.5±5.6	40.8±6.4	43.7±5.4
11. Xiao *et al*., 2021 ^13^	284 subjects Intervention Group (n = 134) Control Group (n = 134)	Wuqinix exercises can improve balance and subjective quality of life in older female patients with knee OA. The therapy significantly improves the clinical symptoms of older female knee OA patients.	WOMAC	98.45±36.01	75.7±20.19	97.95±25.61	94.25±27.73
			Static posture stability	1.75±0.43	1.44±0.18	1.69±0.63	1.63±0.16
12. Kelley Fitzgerald *et al*., 2011 ^42^	183 subjects Intervention Group (n = 91) Control Group (n = 92	Both intervention groups improved in terms of self-reported function and overall change evaluation. The data, however, did not show an additive effect of agility and perturbation training in conjunction with normal exercise treatment.	WOMAC	19.5±12.3	20.3±2.1	19.9±11.9	19.9±1.9
			NPR	4.7±2.6	3.6±0.3	4.4±2.4	3.5±0.35
			Get up and Go test	9.6±2.1	9.1±0.3	9.6±2.3	8.8±0.2
13. Gomiero *et al*., 2017 ^11^	64 subjects Intervention Group (n = 32) Control Group (n = 32)	In individuals with knee OA, resistance training and sensory motor training for the lower limbs appeared to have equivalent advantages on pain and function.	VAS	6.3±0.41	4.6±0.38	6.7±0.45	4.1±0.47
			TUG	9.1±0.59	7.9±0.21	10.5±0.755	8.7±0.505
			Tinetti	24.3±0.83	26.0±0.41	24.1±1.06	26.5±0.38
			WOMAC	36.3±3.58	30.6±3.175	37.8±3.03	29.0±2.86
14. Ojoawo *et al*., 2016 ^27^	45 subjects Group A (n = 23) Group B (n = 22)	Both exercises are helpful, however proprioceptive activities may be more useful than isometric exercises in the treatment of knee OA	Pain	10.71±3.04	3.71±3.40	9.00±3.46	6.50±3.83
			WOMAC	23.71±10.37	10.14±11.48	23.67±8.33	17.67±8.66
15. Jahanjoo *et al*., 2019 ^17^	60 subjects Experimental Group (n = 30) Control Group (n = 30	In those with knee OA, a combination of balance training and physical therapy resulted in greater pain relief and functional capacity development.	VAS	7.30±0.20	3.43±0.23	6.77±0.24	3.83±0.21
			WOMAC	31.40±1.30	22.07±1.29	29.10±1.23	21.17±1.24
			TUG	10.05±0.32	7.61±0.30	10.92±0.32	9.54±0.30
			Fall risk	3.83±0.28	1.90±0.27	4.20±0.34	3.79±0.25
16. Chen *et al*., 2021 ^19^	32 subjects Experimental Group (n = 16) Control Group (n = 16)	In KOA patients, BW is an effective adjunct to normal treatment for reducing pain, improving physical function, and improving static stability. It should be taken into account while developing rehabilitation programmes for people with KOA.	Sway length	594.75±205.13	384.75±106.99	475.44±156.72	383.25±171.88
			Sway area	949.56±552.99	610.50±464.26	629.00±471.67	538.69±420.52
			Proprioception				
			Left	34.63±13.20	29.75±8.07	34.06±10.97	27.06±6.64
			Right	36.25±11.58	28.19±7.90	34.19±14.03	23.88±9.39
			Pain NRS	3.69±0.79	1.56±0.63	3.63±0.96	2.25±0.80
			WOMAC function	14.63±3.56	8.19±1.87	15.00±3.31	12.13±3.20

VAS, Visual Analog Scale; OA, osteoarthritis; WOMAC, Western Ontario and McMaster Universities Arthritis Index; TUG, The Timed Up and Go; BBS, Berg Balance Scale; KAT, Kinesthetic ability trainer; 6MWT, Six-minute walk test; TMT, trail making test; TMFSE, target-matching foot-stepping exercise; AROM, Active range of motion; KT, Kinesio taping; WQXQ, Wu Qin xi Qigong; NPR, numerical pain rating; KOA, knee osteoarthritis; NRS, Numerical pain rating scale; BW, body weight.

*Effect of intervention for multiple groups:* Rogers *et al*., ^29^ conducted a study to compare the efficacy of home-based kinesthesia, balance and agility exercises to resistance exercise, a combination of kinesthesia and resistance exercises, and no exercise. WOMAC pain and physical function significantly improved for all four groups after eight weeks of intervention, with no group differences, although the exercise groups improved more between the midpoint and the eight weeks follow-up, the control group did not. A study conducted by Lin *et al*., ^16^ conducted a study with 89 individuals in which they examined proprioceptive function between computerized proprioception facilitation exercise (CPFE) and closed kinetic chain exercise (CKCE) in subjects with knee OA. After an eight-week exercise intervention, both the CPFE and CKCE groups demonstrated a reduction in knee position error and WOMAC functional scores, as well as increased walking speeds (p = 0.016) on four distinct terrains.

Rahlf *et al*., ^30^ studied the effects of Kinesio taping (KT) on pain and function in patients with knee OA. The WOMAC subscales of pain showed significant differences. However, the effects of KT were more noticeable. Post hoc analysis revealed significant WOMAC differences between the tape and control groups (pain: p = 0.047, function: p = 0.004), the tape and sham groups (pain: p = 0.05, function: p = 0.03), and no difference between the sham and control groups. Trans *et al*., ^26^ studied the effects of whole-body vibration training on proprioception in patients with knee OA. The balance board with built-in vibration (VibF) group improved significantly in the threshold for detection of passive movement (TDPM) when compared to the control group, with a mean difference of -0.59 seconds (95% CI, 1.13 to 0.05; p = 0.0326). When compared to the control group, the balance board with stable vibration platform (VibM) group improved by 0.52 seconds (95% CI, 1.04 to 0.00; p = 0.0511). There was no discernible difference between the VibM and VibF groups in the above study.

Another study, conducted by Braghin *et al*., ^25^ analyzed the effect of exercise on balance and function in people with knee OA. Two of the three groups did physical exercises for 50-60 minutes every day for around eight weeks. The intragroup analysis of the WOMAC questionnaire revealed no differences between the asymptomatic and control groups, whereas the symptomatic group had significantly lower pain and functional outcome scores.

Jan *et al*., ^33^ conducted a study to assess the effects of weight-bearing (WB) and non-weight bearing (NWB) exercise on walking speed, position sensing, and function in people with knee OA. The WOMAC function and walking times on four different terrains improved significantly in the WB group (p = 0.08), but not in the control group. When compared to the control group, both intervention groups improved in WOMAC function (p = 0.08). However, there was no difference between the NWB and WB groups. When compared to the NWB and control groups, the WB group improved more in walking speed on the spongy surface, figure of eight, and positioning error (p = 0.08). The subjects in the NWB exercise group improved their walking speed up and down the stairs. There was no difference in walking speed on level ground between groups, nor was there a difference in walking speed on a figure of eight or a spongy surface, nor was there a difference in reposition error between NWB and the control group.

### Results of syntheses

Meta-analysis was considered for 17 of the included studies, due to heterogeneity in the outcomes. The meta-analysis was done for the common outcomes of VAS, TUG and WOMAC.

### Meta-analysis results

Meta-analysis was conducted for 15 of the 22 included studies. The common outcomes analyzed were VAS scores, WOMAC score and the TUG score.

**Figure 2.**
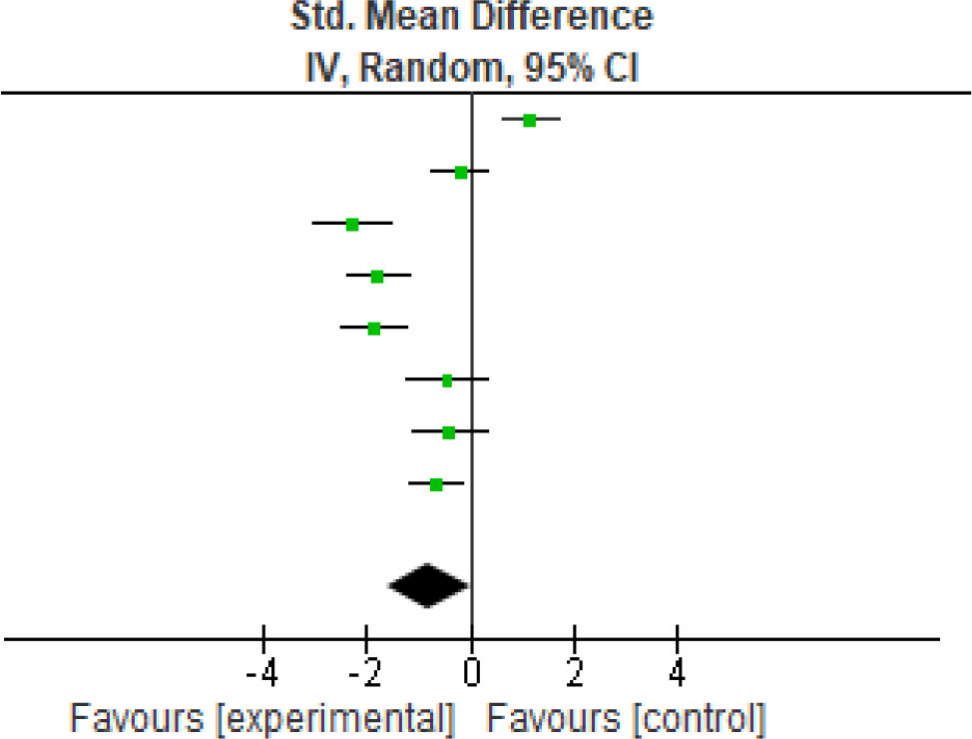
VAS meta-analysis results. VAS, Visual Analog Scale.

For pain, ( Figure 2) eight studies were analyzed for the VAS scores, pre-and post-intervention. Heterogeneity [ *I* ^2^] was 92% (p < 0.05). The mean difference was -0.79 with (95% CI, -1.59 to 0.01) for the intervention versus the control group ( Table 3).

**Table 3.** VAS meta-analysis results. VAS, Visual Analog Scale.

Study or subgroup	Experimental			Control			Weight	Std. Mean Difference
	Mean	SD	Total	Mean	SD	Total		IV, Random, 95% CI
Gomiero *et al*., 2017	4.6	0.38	32	4.1	0.47	32	12.8%	1.16 [0.62, 1.69]
Hussein *et al*., 2015	40.0	14.23	38	42.8	15.21	21	12.8%	-0.19 [-0.72, 0.34]
Cho *et al*., 2015	50.0	7.7	23	67.2	7.2	23	12.1%	-2.27 [-3.02, -1.51]
Jahanjoo *et al*., 2019	3.43	0.23	30	3.83	0.21	30	12.6%	-1.79 [-2.40, -1.19]
Kuru Colak *et al*., 2017	39.58	4.39	33	50.09	6.99	23	12.5%	-1.85 [-2.49, -1.21]
Oh *et al*., 2020	4.2	1.7	13	4.9	1.4	13	12.0%	-0.44 [-1.21, 0.34]
Reza *et al*., 2018	4.6	1.91	15	5.4	2.09	15	12.2%	-0.39 [-1.11, 0.33]
Tunay *et al*., 2010	1.46	2.04	30	2.8	2.02	30	12.9%	-0.65 [-1.17, -0.13]
Total (95% CI)			214			187	100.0%	-0.79 [-1.59, 0.01]

Heterogeneity: Tau ^2^ = 1.22; Chi ^2^ = 92.84, df = 7 (p < 0.00001); *I* ^2^ = 92%.

Test for overall effect: Z = 1.95 (p = 0.05).

**Figure 3.**
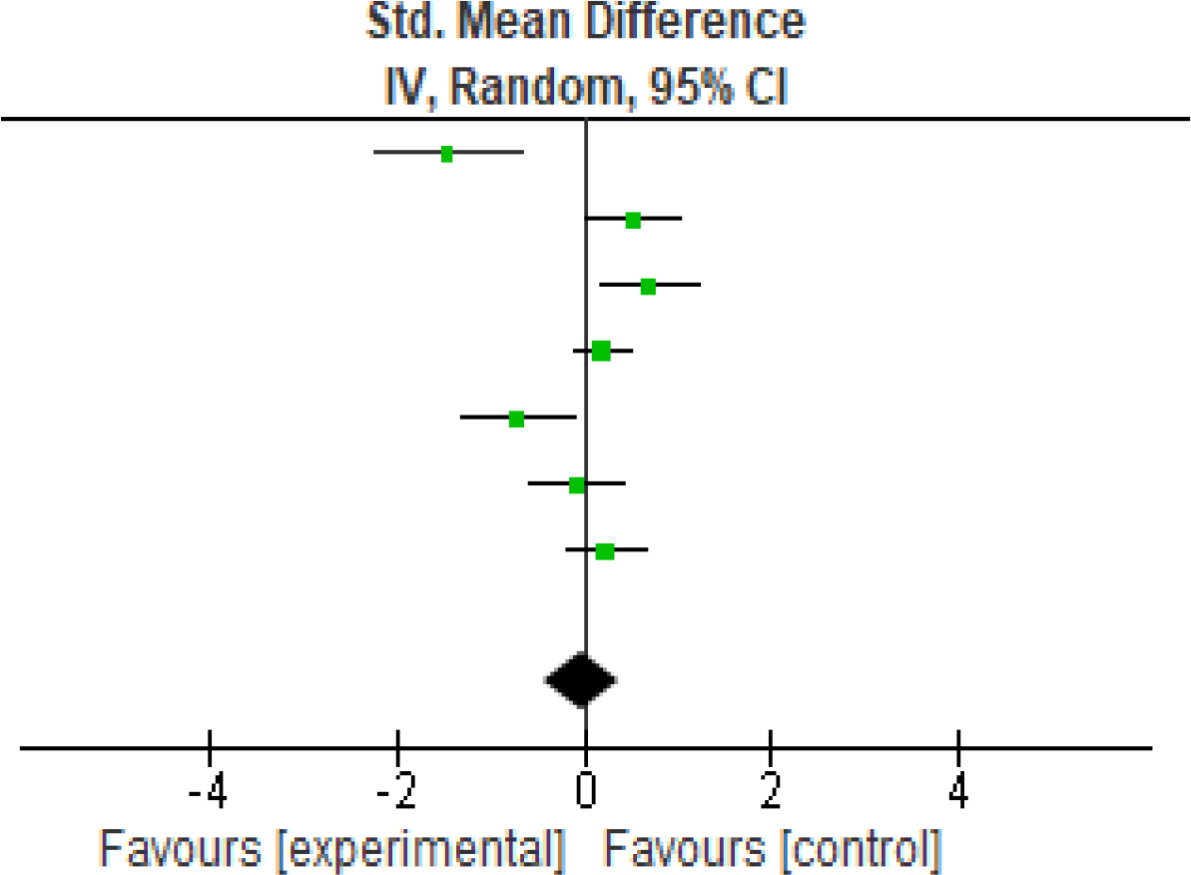
WOMAC meta-analysis results. WOMAC, Western Ontario and McMaster Universities Arthritis Index.

For functional outcome, seven studies were analyzed using the WOMAC ( Figure 3) scores the heterogeneity [ *I* ^2^] was 81% (p < 0.0001). The mean difference was -0.02 (95% CI, -0.44 to 0.40) for the intervention against the control group ( Table 4).

**Table 4.** WOMAC meta-analysis results. WOMAC, Western Ontario and McMaster Universities Arthritis Index.

Study or subgroup	Experimental			Control			Weight	Std. Mean Difference
	Mean	SD	Total	Mean	SD	Total		IV, Random, 95% CI
Chen *et al*., 2021	8.19	1.87	16	12.13	3.2	16	11.1%	-1.47 [-2.26, -0.67]
Gomiero *et al*., 2017	30.6	3.18	32	29.0	2.86	32	14.6%	0.52 [0.02, 1.02]
Jahanjoo *et al*., 2019	22.07	1.29	30	21.17	1.24	30	14.3%	0.70 [0.18, 1.22]
Kelley Fitzerald *et al*., 2011	20.3	2.1	91	19.9	1.9	92	16.9%	0.20 [-0.09, 0.49]
Ojoawo *et al*., 2016	10.14	11.48	23	17.67	8.66	22	13.3%	-0.73 [-1.33, -0.12]
Tunay *et al*., 2010	5.45	3.76	30	5.69	2.84	30	14.5%	-0.07 [-0.58, 0.44]
Xiao *et al*., 2020	20.7	8.7	40	18.8	7.4	45	15.4%	0.23 [-0.19, 0.66]
Total (95% CI)			262			267	100.0%	-0.02 [-0.44, 0.40]

Heterogeneity: Tau ^2^ = 0.25; Chi ^2^ = 31.26, df = 6 (p < 0.0001); *I* ^2^ = 81%.

Test for overall effect: Z = 0.11 (p = 0.91).

**Figure 4.**
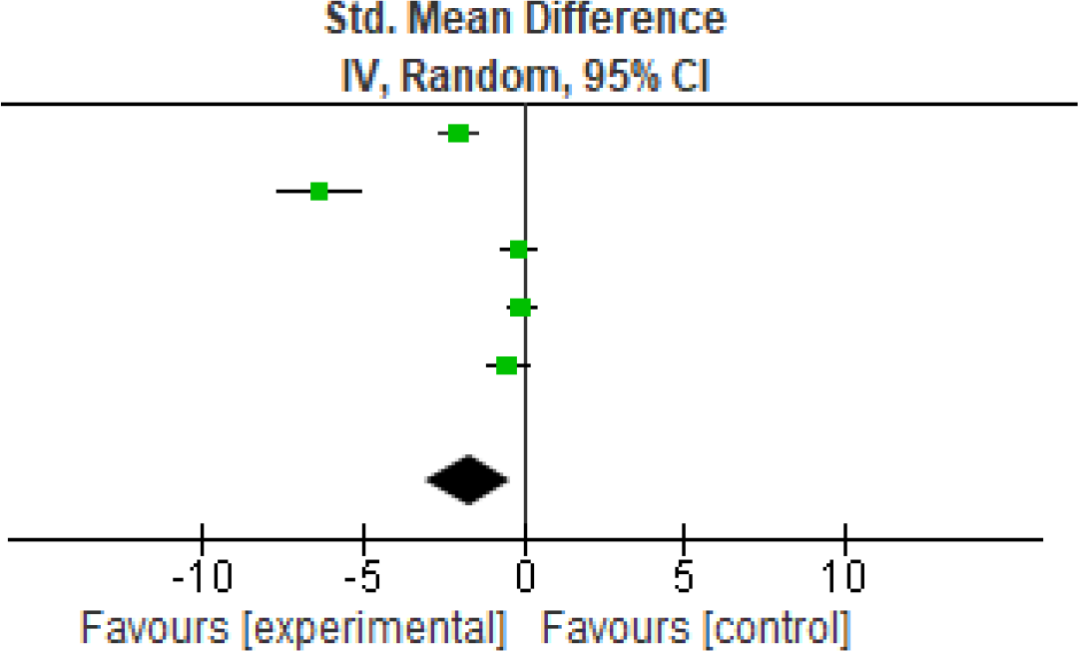
TUG meta-analysis results. TUG, The Timed Up and Go.

Another functional parameter was analyzed using the TUG ( Figure 4) score. Five studies were analyzed and the heterogeneity [ *I* ^2^] was 95% (p < 0.0001). The mean difference was -1.71 (95% CI, -3.09 to -0.33) for the intervention against the control group ( Table 5).

**Table 5.** TUG meta-analysis results. TUG, The Timed Up and Go.

Study or subgroup	Experimental			Control			Weight	Std. Mean Difference
	Mean	SD	Total	Mean	SD	Total		IV, Random, 95% CI
Gomiero *et al*., 2017	7.9	0.21	32	8.7	0.51	32	20.4%	-2.03 [-2.64, -1.42]
Jahanjoo *et al*., 2019	7.61	0.3	30	9.54	0.3	30	17.9%	-6.35 [-7.63, -5.07]
Tunay *et al*., 2010	5.19	1.05	30	5.39	1.46	30	20.6%	-0.16 [-0.66, 0.35]
Xiao *et al*., 2020	7.7	2.7	40	7.9	2.2	45	20.8%	-0.08 [-0.51, 0.35]
Hiyama *et al*., 2012	12.0	1.5	20	13.0	2.2	20	20.3%	-0.52 [-1.15, 0.11]
Total (95% CI)			152			157	100.0%	-1.71 [-3.09, -0.33]

Heterogeneity: Tau ^2^ = 2.34; Chi ^2^ = 105.66, df = 4 (p < 0.00001); *I* ^2^ = 96%.

Test for overall effect: Z = 2.42 (p = 0.02).

According to the findings of the included studies and their meta-analysis, balance-based exercises help to reduce pain and improve functional outcomes in people with balance alteration following knee OA. The above values are displayed in a small confidence interval range, indicating the analyses’ validity and sensitivity, as well as the significant influence. Furthermore, the random-effects model used provided accurate results by using sample size and standard error. The meta-analysis likewise comes up with a positive conclusion balance-based exercise. Even though the key outcome measures of VAS, WOMAC and TUG scores were homogeneous, the analyzed studies differed in the mode and duration of intervention and hence a meta-analysis was conducted using the random-effects model.

### Reporting biases

The studies in included our review were of moderate to good methodological quality, however, risk of bias was noted in terms, of blinding of subjects, therapist and assessors. A detailed description of the same is given in Table 1.

### Certainty of evidence

Certainty of evidence is low to moderate due to the quality of the included studies and higher heterogeneity.

## Discussion

Our systematic analysis included 22 trials that assessed the effectiveness of balance exercises on functional outcomes and pain in people with knee OA. We were only able to include 13 publications in the meta-analysis due to the variety of the therapies and outcomes investigated. Several therapies, including strength training, Wu Qin xi Qigong (WQXQ), Wuqinix, visual reality feedback exercises, and agility training, dramatically improved balance, reduced discomfort, and improved functional outcomes in people with knee OA. Functional outcomes examined using sit to stand and balance measured using the BBS did not show statistically significant improvement in the experimental group among the trials included in the review. Functional results based on gait speed, on the other hand, TUG, ^11^^,^^14^^,^^17^^,^^20^^,^^31^^,^^32^ WOMAC, ^11^^–^^14^^,^^17^^,^^20^^,^^27^^,^^31^ VAS, ^11^^,^^12^^,^^15^^,^^17^^–^^21^^,^^23^^,^^24^^,^^35^ and six minute walk test (6MWT). ^14^^,^^23^ The experimental group outperformed the control group significantly.

The meta-analysis comprised 13 articles that employed VAS, WOMAC, and TUG as outcome measures. When compared to regular routine rehabilitation exercises, VAS and TUG outcomes for subjects assigned to the investigational group showed superior results, indicating that rehabilitation strategies improved knee joint proprioception and balance, indicating efficacy for the intervention, whereas WOMAC showed no significant difference between the experimental and control group.

Previous studies have demonstrated that pain can produce inflammatory chemicals that can sensitize a significant number of nerve terminals. Afferent nerve impulses from small-diameter groups three and four (pain) and large-diameter group two (proprioceptive) may fire abnormally, resulting in aberrant JPS and muscle spindle activity. ^36^^,^^37^ It was theorized that implementing pain-relieving exercise therapies in such situations may improve JPS and thereby enhance balance by reducing stiffness, increasing joint lubrication and enhancing muscle strength. ^38^ The aforementioned mechanism may explain the meta-analysis difference in VAS between experimental and control groups.

Agility and perturbation training exercises were utilized as the mode of intervention in studies by Fitzgerald *et al*., ^12^ and Rogers *et al*. ^29^ The reasoning for integrating agility and perturbation programs was to expose the participants to demanding movements such as quick changes in direction, and short stop and starts to improve actions during routine activity. The aforementioned strategies may be beneficial for younger and athletic people. ^12^ In older adults with knee OA, the requirements of agility and daily challenges to balance may be less frequent when compared to younger athletes and therefore, inclusion of agility and perturbation techniques into exercise regimens may have no beneficial effect in enhancing knee stability and general function among older individuals. Among all the studies, the study conducted by Fitzgerald *et al*., ^12^ had the largest sample size compared to the rest, and excluded participants based on the risk for fall and need for assistive devices for ambulation. ^12^

Cho *et al*., ^18^ conducted a study that included taping as an intervention and determined that taping improved balance and anticipated that the elastic property of the tape and its application under tension leads to mobilization of the skin during movements and increases blood and lymph circulation. The aforementioned process is believed to have a direct impact on pain perception. ^39^^,^^40^

### Limitations and future scope

As the current systematic review includes data from studies with varied sample sizes, the treatment impact could be overestimated. Additionally, comparing outcome measures was difficult due to the diversity of the assessment procedures utilized between studies. A thorough literature search conducted to expand empirical understanding yielded various areas of recommendation. To begin, a larger, suitably powered RCT should be done to offer critical data on the type and dosage of exercises needed. Second, we advocate conducting another systematic review to look into other factors that influence balance, such as OA symptoms (functional limitation, pain, stiffness), quality-of-life domains, and other functional measures.

## Conclusions

Our review’s findings are persuasive that a basic balance training program may improve physical function, reduce pain, and increase physical activity levels in individuals with knee OA. Although proprioceptive and balance exercises reduced pain and TUG considerably, there was no noticeable difference in WOMAC ratings among individuals. Further research into various therapy techniques in a bigger population is required.

## Data availability

### Underlying data

All data underlying the results are available as part of the article and no additional source data are required.

### Extended data

Open Science Framework: Effectiveness of balance training on pain and functional outcomes in knee osteoarthritis - a systematic review and meta-analysis. https://doi.org/10.17605/OSF.IO/4H7TQ. ^43^

This project contains the following extended data:

- DATA EX TABLE.docx (Effects of intervention of the included studies)
- Key Word F1000.docx (Key search terms)
- Protocol OSF F1000 (1).docx
- TITLE PAGE.docx

Data are available under the terms of the Creative Commons Zero “No rights reserved” data waiver (CC0 1.0 Public domain dedication).

### Reporting guidelines

Open Science Framework: PRISMA checklist for ‘Effectiveness of balance training on pain and functional outcomes in knee osteoarthritis: A systematic review and meta-analysis’. https://doi.org/10.17605/OSF.IO/4H7TQ. ^43^

Data are available under the terms of the Creative Commons Zero “No rights reserved” data waiver (CC0 1.0 Public domain dedication).
