# Supplementary material for: Effectiveness of balance training on pain and functional outcomes in knee osteoarthritis: A systematic review and meta-analysis
Source: F1000Res. 2023 Oct 13;11:598. Originally published 2022 Jun 1. [Version 2] doi: 10.12688/f1000research.111998.2 (PMC10912788; doi:10.12688/f1000research.111998.2)
Supplement: Peer review history

# Reviewer report: Reviewer response for version 2

**DOI:** 10.5256/f1000research.156648.r247724

**Published:** 2024-03-04

**Authors:** Vijay kage

**Affiliations:** 1 Department of Orthopaedic Physiotherapy, KAHER Institute of Physiotherapy, Belagavi, Karnataka, India

**Major Modifications**

 1. The rationale of the study is not specifying the need for evaluating balance training on the functional outcomes and pain, this requires mor specification and justification as very less is written on the predicted title

 2. In Introduction more is mentioned about proprioception, but this is not considered as the aim of the study

 3. In methodology inclusion criteria specifies about RCT comparing balance exercises with conventional but as the aim specifies about pain and functional outcomes it is not considered as part of inclusion

 4. Inclusion criteria the 2nd  inclusion mentioned as age group 40 and above while the 3rd inclusion point is on any age (Kindly justify the 2nd inclusions). Also the 3rd inclusion mentions the severity of OA (Kindly justify the same). The article requires minor explanation. Rest the article is written well

 5. The search strategies for the systematic review is not mentioned like using Boolean search using AND/ OR/ NOT

 6. Very less is mentioned about the outcomes used in various studies as that was the primary purpose of this systematic review more must be written regarding the same

**Minor Modifications**

 1. Short forms are not mentioned in full detail like TUG (This appears first in the methodology but is been mentioned in discussion)

 2. The systematic review mentions more about WOMAC, TUG and not about the other functional outcomes and also pain

Are the rationale for, and objectives of, the Systematic Review clearly stated?

Yes

Is the statistical analysis and its interpretation appropriate?

Yes

Are sufficient details of the methods and analysis provided to allow replication by others?

Yes

Are the conclusions drawn adequately supported by the results presented in the review?

Yes

Reviewer Expertise:

Musculoskeletal research, Sports and Rehabilitation

I confirm that I have read this submission and believe that I have an appropriate level of expertise to confirm that it is of an acceptable scientific standard.

# Reviewer report: Reviewer response for version 2

**DOI:** 10.5256/f1000research.156648.r214992

**Published:** 2023-12-15

**Authors:** Sohrab Ahmad Khan

**Affiliations:** 1 Department of Physiotherapy, Jamia Hamdard, New Delhi, India

Now, it is much better than the previously written article.

Are the rationale for, and objectives of, the Systematic Review clearly stated?

Partly

Is the statistical analysis and its interpretation appropriate?

Partly

Are sufficient details of the methods and analysis provided to allow replication by others?

Partly

Are the conclusions drawn adequately supported by the results presented in the review?

No

Reviewer Expertise:

Musculoskeletal Physiotherapy, Biomarkers in Osteoarthritis, Orthopedic physiotherapy

I confirm that I have read this submission and believe that I have an appropriate level of expertise to confirm that it is of an acceptable scientific standard, however I have significant reservations, as outlined above.

# Reviewer report: Reviewer response for version 1

**DOI:** 10.5256/f1000research.123724.r179304

**Published:** 2023-08-23

**Authors:** Sohrab Ahmad Khan

**Affiliations:** 1 Department of Physiotherapy, Jamia Hamdard, New Delhi, India

Explain the rationale of the research in detail, with need of doing this systematic review as there is ample number of studies and reviews already done. What does your article adds to the existing pool of literature.

 In the methodology section, Inclusion and exclusion criteria needs to be explained well, Did you include all paid full text articles in all languages? Also mention about paid articles included or not/ open access only 

 In 22 years, with such a variety of databases, only 840 articles were available? In search strategy, only online databases are mentioned. In Prisma flowchart, registers show 5 records screened. Edit the search strategy accordingly.

 The search strategy should be mentioned. Write the exact search strategy that you used using Boolean phrases. 

 Discussion section needs to be rephrased in a better understandable way.

 Conclusion needs to be as per the aim of the study.

Are the rationale for, and objectives of, the Systematic Review clearly stated?

Partly

Is the statistical analysis and its interpretation appropriate?

Partly

Are sufficient details of the methods and analysis provided to allow replication by others?

Partly

Are the conclusions drawn adequately supported by the results presented in the review?

No

Reviewer Expertise:

Musculoskeletal Physiotherapy, Biomarkers in Osteoarthritis, Orthopedic physiotherapy

I confirm that I have read this submission and believe that I have an appropriate level of expertise to confirm that it is of an acceptable scientific standard, however I have significant reservations, as outlined above.

# Response

**Published:** 2023-09-26

**Authors:** Tina Dias

**Affiliations:** Kasturba Medical College Hospital, India

Dear Reviewer,

 We thank the reviewer and the editorial team for taking the time to conduct an in-depth review of our article. We thank you for the appreciation you have given us regarding this systematic review. We acknowledge the corrections and will upload them in the corrected version of the document

# Reviewer report: Reviewer response for version 1

**DOI:** 10.5256/f1000research.123724.r154625

**Published:** 2022-11-11

**Authors:** Pratik Phansopkar

**Affiliations:** 1 Department of Musculoskeletal Physiotherapy, Ravi Nair Physiotherapy College, DDatta Meghe Institute of Medical Sciences, Wardha, India

This systematic review and meta-analysis included 22 randomized controlled trials for systematic review and 17 studies for meta-analysis assessing the impact of balance exercises on pain and functional activities in patients with knee osteoarthritis. 

 In the method section, the author is requested to add the lower age limit of the patients included in the randomized controlled trial which was then included in this systematic review and meta-analysis.

 The content included in the study selection and inclusion and exclusion criteria is repeating itself. Kindly check for a better content presentation.

 The authors have mentioned 15 studies included in the meta-analysis result section however in the discussion section they have mentioned the inclusion of 13 publications. Therefore, the authors are requested to clarify the inclusion of articles in the meta-analysis.

Are the rationale for, and objectives of, the Systematic Review clearly stated?

Yes

Is the statistical analysis and its interpretation appropriate?

Yes

Are sufficient details of the methods and analysis provided to allow replication by others?

Yes

Are the conclusions drawn adequately supported by the results presented in the review?

Yes

Reviewer Expertise:

My area of expertise includes Musculoskeletal Physiotherapy, Physiotherapy Research, Advances in Physiotherapeutics.

I confirm that I have read this submission and believe that I have an appropriate level of expertise to confirm that it is of an acceptable scientific standard.

# Response

**Published:** 2022-11-12

**Authors:** Tina Dias

**Affiliations:** Kasturba Medical College Hospital, India

Dear Reviewer,

 We thank the reviewer and the editorial team for taking the time to conduct an in-depth review of our article. We thank you for the appreciation you have given us regarding this systematic review. We acknowledge the corrections and will upload them in the corrected version of the document 

 REVIEWER 1 

 1) In the method section, the author is requested to add the lower age limit of the patients included in the randomized controlled trial which was then included in this systematic review and meta-analysis. 

 RESPONSE - Under the description of the included studies sub-topic (Participant description) The age limit of the included subjects in the RCT has been mentioned. 

 2)The content included in the study selection and inclusion and exclusion criteria is repeating itself. Kindly check for a better content presentation.

 RESPONSE - The correction will be made in the new uploaded version of the article.

 3) The authors have mentioned 15 studies included in the meta-analysis result section however in the discussion section they have mentioned the inclusion of 13 publications. Therefore, the authors are requested to clarify the inclusion of articles in the meta-analysis.

 RESPONSE - The correction will be made in the new uploaded version of the article.
